# K48-linked KLF4 ubiquitination by E3 ligase Mule controls T-cell proliferation and cell cycle progression

**DOI:** 10.1038/ncomms14003

**Published:** 2017-01-13

**Authors:** Zhenyue Hao, Yi Sheng, Gordon S. Duncan, Wanda Y. Li, Carmen Dominguez, Jennifer Sylvester, Yu-Wen Su, Gloria H.Y. Lin, Bryan E. Snow, Dirk Brenner, Annick You-Ten, Jillian Haight, Satoshi Inoue, Andrew Wakeham, Alisha Elford, Sara Hamilton, Yi Liang, Juan C. Zúñiga-Pflücker, Housheng Hansen He, Pamela S. Ohashi, Tak W. Mak

**Affiliations:** 1 The Campbell Family Institute for Breast Cancer Research, University Health Network, Toronto, Ontario, Canada M5G 2M9; 2 Princess Margaret Cancer Centre, University Health Network, Toronto, Ontario, Canada M5G 2M9; 3 Department of Medical Biophysics, University of Toronto, Toronto, Ontario, Canada M5G 2C1; 4 The Donnelly Centre for Cellular and Biomolecular Research, Banting and Best Department of Medical Research, and Department of Molecular Genetics, University of Toronto, 160 College Street, Toronto, Ontario, Canada M5S3E1; 5 Department of Biology, York University, Toronto, Ontario, Canada M3J 1P3; 6 Immunology Research Center, National Health Research Institutes, Zhunan, Miaoli County 35053, Taiwan; 7 Department of Infection and Immunity, Experimental and Molecular Immunology, Luxembourg Institute of Health, 29, rue Henri Koch, Esch-sur-Alzette L-4354, Luxembourg; 8 Odense Research Center for Anaphylaxis (ORCA), Department of Dermatology and Allergy Center, Odense University Hospital, University of Southern Denmark, Odense DK-5000 Denmark; 9 Department of Immunology, University of Toronto, Toronto, Ontario, Canada M5G 2C1; 10 Sunnybrook and Women's College Health Sciences Centre, Toronto, Ontario, Canada M4N 3M5

## Abstract

T-cell proliferation is regulated by ubiquitination but the underlying molecular mechanism remains obscure. Here we report that Lys-48-linked ubiquitination of the transcription factor KLF4 mediated by the E3 ligase Mule promotes T-cell entry into S phase. Mule is elevated in T cells upon TCR engagement, and *Mule* deficiency in T cells blocks proliferation because KLF4 accumulates and drives upregulation of its transcriptional targets E2F2 and the cyclin-dependent kinase inhibitors p21 and p27. T-cell-specific *Mule* knockout (TMKO) mice develop exacerbated experimental autoimmune encephalomyelitis (EAE), show impaired generation of antigen-specific CD8^+^ T cells with reduced cytokine production, and fail to clear LCMV infections. Thus, Mule-mediated ubiquitination of the novel substrate KLF4 regulates T-cell proliferation, autoimmunity and antiviral immune responses *in vivo*.

## Abstract (web summary)

The E3 ligase Mule has been previously reported to be essential for B cell development and function by modulating p53 ubiquitination and degradation. Here Hao *et al*. identify KLF4 as a novel ubiquitination target of Mule and show it controls T cell proliferation and autoimmunity.

Engagement of the T-cell receptors (TCR) of a mature naive T cell triggers signalling leading to activation of kinases and transcription factors^1^,^2^. Because dysregulation of these pathways impairs homoeostasis and immune responses, and can lead to lymphoma or autoimmunity, they are tightly regulated at multiple levels. E3 ligase-mediated ubiquitination is one of these essential regulatory mechanisms^3^,^4^,^5^,^6^. TCR signalling and peripheral T-cell tolerance are known to be modulated via the ubiquitination of upstream elements, including TCRζ by Itch^7^, ZAP70 by the Cbl family and Nrdp1 (ref. 5), and phosphatidylinositol-3 kinase by Cbl-b^8^. However, whether E3 ligases are also involved in targeting downstream elements such as transcription factors, drivers of differentiation and cell cycle regulators remains unclear.

The transcription factors KLF4 and ELF4 negatively regulate CD8^+^ T-cell proliferation and differentiation through their control of cyclin-dependent kinases (CDK) and their inhibitors (CDKI)^9^,^10^. KLF4 protein diminishes in response to TCR engagement, leading to downregulation of CDKI such as p21 and p27 and the entrance of CD8^+^ T cells into the cell cycle. CD8^+^ T cells lacking either *klf4* or *elf4* hyperproliferate upon TCR engagement^9^,^10^. In CD4^+^ T cells, KLF4 binds to the IL17a promoter and drives Th17 differentiation independently of RORγt^11^,^12^. Accordingly, T-cell-specific *Klf4* knockout (KO) mice are resistant to induction of experimental autoimmune encephalomyelitis (EAE) due to impaired Th17 differentiation. KLF4 also drives transcription of E2F2, which acts as a transcriptional repressor inhibiting cell cycle entry^13^. Like KLF4 deficiency, deletion of *e2f2* in mice enhances T-cell proliferation and leads to autoimmunity^14^.

Mule (Mcl-1 Ubiquitin Ligase E3, also called Huwe1, ArfBP1 and Lasu1) is a HETC domain-containing E3 ligase that mediates ubiquitination of a broad range of substrates, including cMyc^15^, Mcl-1 (ref. 16) and p53 (ref. 17). cMyc influences T-cell activation and proliferation both directly and indirectly through control of transcriptional targets and metabolic reprogramming^18^,^19^,^20^, and by modulating the expression of cell cycle regulators^21^. Mcl-1 is critical for T-cell development and mature T-cell survival due to its anti-apoptotic effects^16^,^22^. We previously showed that Mule-mediated polyubiquitination and degradation of p53 is required for B-cell development, homoeostasis and humoral immune responses^23^. To examine Mule's role in T-cell biology *in vivo*, we generated T-cell-specific Mule-deficient mice and studied the consequences of Mule inactivation in these mutants and their T cells. *In vivo*, these animals develop severe EAE and show impaired antiviral immune responses. *In vitro*, we identify KLF4 as a novel Mule substrate and demonstrate that Mule-mediated regulation of KLF4 controls TCR-mediated T-cell proliferation at the level of cell cycle entry.

## Results

### Loss of Mule leads to impaired T-cell homoeostasis

**Figure 1:**
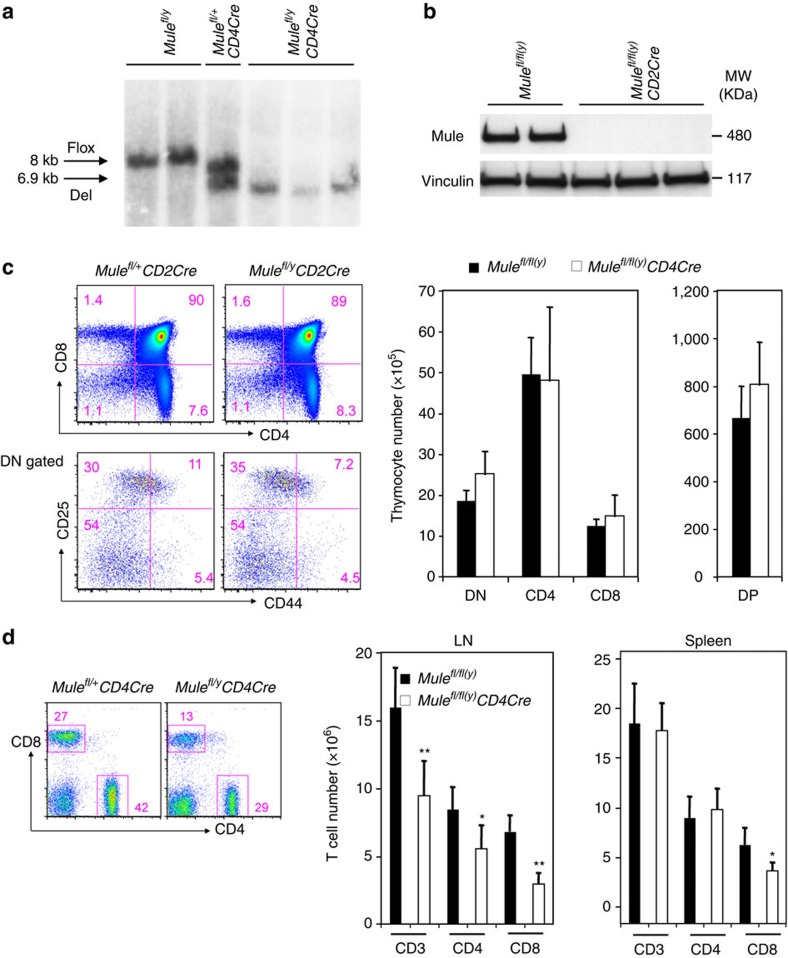
Impaired T-cell homoeostasis in TMKO mice. (**a**) Southern blot of genomic DNA from thymocytes of *Mule*^*fl/y*^, *Mule*^*fl/+*^*CD4Cre* and *Mule*^*fl/y*^*CD4Cre* (TMKO) mice indicating the floxed and deleted *Mule* alleles. (**b**) Immunoblot (IB) of Mule protein in thymocytes of *Mule*^*fl/fl(y)*^ and *Mule*^*fl/fl(y)*^*CD2Cre* (TMKO) mice. Vinculin, loading control. (**c**) Top left: FCM analysis of CD4 versus CD8 expression by thymocytes from control and TMKO mice. Numbers in quadrants are percentages of gated lymphocytes. Bottom left: FCM analysis of CD25 versus CD44 expression by DN-gated, lineage (CD4, CD8, TCRγδ, B200, NK1.1, Gr1 and TER 119) negative cells. Percentages of DN1 (CD44^+^CD25^−^), DN2 (CD44^+^CD25^+^), DN3 (CD44^−^CD25^+^) and DN4 (CD44^−^CD25^−^) thymocytes among the gated DN population are indicated. Right: numbers of thymocytes in the indicated subsets: DN (CD4^−^CD8^−^), CD4 single positive, CD8 single positive and DP (double positive, CD4^+^CD8^+^). Results are representative of 3–5 mice/genotype. (**d**) Left: FCM analysis of CD4 versus CD8 expression by gated LN T cells from control and TMKO mice. Middle and right: quantitation of CD3^+^, CD4^+^ and CD8^+^ T-cell numbers in LN and spleen of control and TMKO mice. Data are the mean±s.d. (*n*=6); *P<0.5, **P<0.05. *P* values were calculated with one-sided Student's *t*-test. Results are representative of two to three independent experiments involving three to four mice per genotype.

To achieve *Mule* ablation in a T-cell-specific manner, we bred conditional *Mule*^*fl/fl(y)*^ mutant mice^23^ to either *CD4Cre* transgenic (Tg) mice in which *Cre* is controlled by a *CD4* mini-gene^24^, or *CD2Cre* Tg mice in which *Cre* is regulated by the human *CD2* promoter^25^. Southern blotting and immunoblotting analyses of the resulting *Mule*^*fl/fl(y)*^*CD4Cre* or *Mule*^*fl/fl(y)*^*CD2Cre* mice (collectively, TMKO mice) confirmed efficient Mule deletion in the thymus (Fig. 1a,b). Flow cytometric (FCM) profiling of immunostained thymocytes from TMKO mice showed that the CD4^+^ versus CD8^+^ populations, as well as the CD25^+^ versus CD44^+^ subsets among CD4^−^CD8^−^ (double negative; DN) thymocytes, were comparable to those in *Mule*^*fl/fl(y)*^ controls (Fig. 1c, left). The total cellularities of the CD4^−^CD8^−^ DN, CD4^+^ single positive, CD8^+^ single positive and CD4^+^CD8^+^ (double positive) compartments in TMKO mice were also similar to those in controls (Fig. 1c, right). However, TMKO lymph nodes (LN) showed significant decreases in total CD3^+^ T cells as well as in the CD4^+^ and CD8^+^ subsets (Fig. 1d, middle). In the spleen, TMKO mice exhibited reduced CD8^+^ T-cell numbers but normal total and CD4^+^ T-cell numbers (Fig. 1d, right). To examine the emigration of T cells from the thymus, control and TMKO mice were supplied with BrdU-containing drinking water for 3 days. In TMKO mice, both the CD4^+^BrdU^lo^ and CD8^+^BrdU^lo^ populations, which represent T cells that have recently immigrated from the thymus^26^, were significantly reduced compared with controls (Supplementary Fig. 1a,b). However, the CD4^+^BrdU^hi^ and CD8^+^BrdU^hi^ populations were equivalent in TMKO and control mice. The defective thymic output in TMKO mice may be partially attributed to the lower level of CD44 expression by naive CD4^+^ and CD8^+^ T cells in these animals. These results suggest that Mule is dispensable for thymic T-cell development but important for thymic emigration and thus peripheral T-cell maintenance.

### TMKO T cells show poor proliferation upon TCR engagement

**Figure 2:**
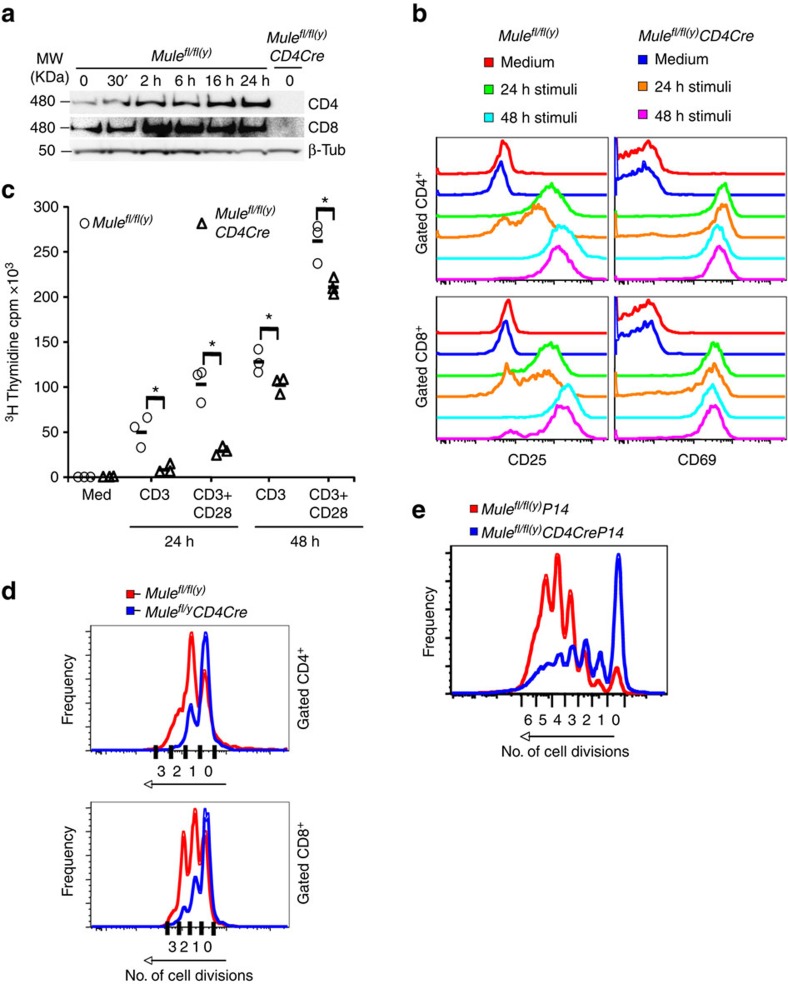
Impaired proliferation of *Mule*-deficient T cells. (**a**) IB to detect Mule protein in purified control and TMKO CD4^+^ and CD8^+^ T cells that were left untreated (0) or stimulated with anti-CD3 plus anti-CD28 Abs for the indicated times. beta-Tub, loading control. (**b**) FCM analysis of CD25 and CD69 expression by gated control or TMKO CD4^+^ and CD8^+^ T cells at 24 and 48 h after stimulation with medium alone or medium containing anti-CD3/28 Abs (stimuli). (**c**) Quantitation of [^3^H]thymidine incorporation by purified control and TMKO LN T cells that were cultured for 24 h and 48 h in medium alone (med), or in medium containing anti-CD3 (1 μg ml^−1^), or anti-CD3 (1 μg ml^−1^) plus anti-CD28 (1 μg ml^−1^). Results are the mean±s.d. (*n*=3); *P<0.5. *P* values were calculated with one-sided Student's *t*-test. (**d**) FCM analysis of purified control and TMKO T cells that were labelled with VCT to monitor cell divisions and stimulated with anti-CD3/28 Abs for 48 h. Each peak indicates one cell division in gated CD4^+^ (top) and CD8^+^ (bottom) T cells. (**e**) FCM analysis of purified CD8^+^ T cells from *Mule*^*fl/fl(y)*^*P14* and *Mule*^*fl/fl(y)*^*CD4CreP14* mice that were VCT-labelled and stimulated with GP_33–41_ peptide for 72 h. Cell divisions were analysed as in **d**. Results are representative of two to three independent experiments involving two to six mice per genotype.

We next examined changes to normal Mule protein levels upon T-cell activation by stimulating purified WT CD4^+^ and CD8^+^ mature T cells with plate-bound anti-CD3 plus anti-CD28 antibodies (Abs). Mule was rapidly elevated in response to these stimuli and maintained at high levels from 2–24 h post-stimulation (Fig. 2a and Supplementary Fig. 4a and b), suggesting that Mule might be involved in TCR-mediated T-cell activation and proliferation. Mule was undetectable in resting TMKO T cells and also in anti-CD3/CD28-treated TMKO T cells at 24 h post-stimulation. FCM analysis showed that, while all WT CD4^+^ T cells had upregulated CD25 by 1 day post-TCR stimulation, only 70% of TMKO CD4^+^ T cells did so, and this level of CD25 upregulation was ∼2-fold lower than in controls (Fig. 2b, top). By 48 h post-stimulation, CD25 upregulation by TMKO CD4^+^ T cells had increased substantially but was still lower than that in WT T cells. TMKO CD8^+^ T cells also showed reduced CD25 upregulation at 24 h post-TCR stimulation but had partially caught up by 48 h (Fig. 2b, bottom left). In contrast to CD25, TMKO CD4^+^ and CD8^+^ T cells upregulated CD69 just as efficiently as WT controls (Fig. 2b, right). Thus, TCR-stimulated activation of TMKO T cells is compromised during the early activation phase (within 24 h) but recovers to some extent by 48 h. Confirming this hypothesis, measurement of thymidine incorporation by TMKO T cells at 24 h after treatment with either anti-CD3/CD28 Abs, or with phorbol myristate acetate (PMA) plus calcium ionophore (Iono), showed defective proliferation compared with controls (Fig. 2c).

To assess whether the early proliferation defect in TMKO T cells was associated with altered cell division, we labelled WT and TMKO T cells with violet cell tracker (VCT), activated them with anti-CD3/28 Abs, and followed their mitosis by FCM. About twofold more TMKO CD4^+^ and CD8^+^ T cells failed to divide compared with WT cultures (CD4^+^ TMKO versus WT: 74% versus 31%; CD8^+^: 64% versus 32%) (Fig. 2d). TMKO T cells that did cycle underwent one less cell division than controls. Thus, TMKO T cells have a defect in cell division triggered by TCR engagement.

To determine whether the proliferation defect in TMKO T cells was associated with specific antigen, we bred TMKO mice to P14 transgenic mice expressing a TCR specific for the lymphocytic choriomeningitis virus (LCMV) glycoprotein peptide 33–41 (GP33) presented by MHC I molecule H-2D^b^ (ref. 27). We labelled WT and TMKO P14 Tg T cells with VCT, activated them with GP33 peptide and followed their cell division by FCM. About 34% of CD8^+^ TMKO P14 T cells failed to divide compared with ∼5% of controls (Fig. 2e). Thus, *Mule*-deficient CD8^+^ T cells do not proliferate efficiently in response to stimulation by either anti-CD3/28 Abs or specific antigen.

**Figure 3:**
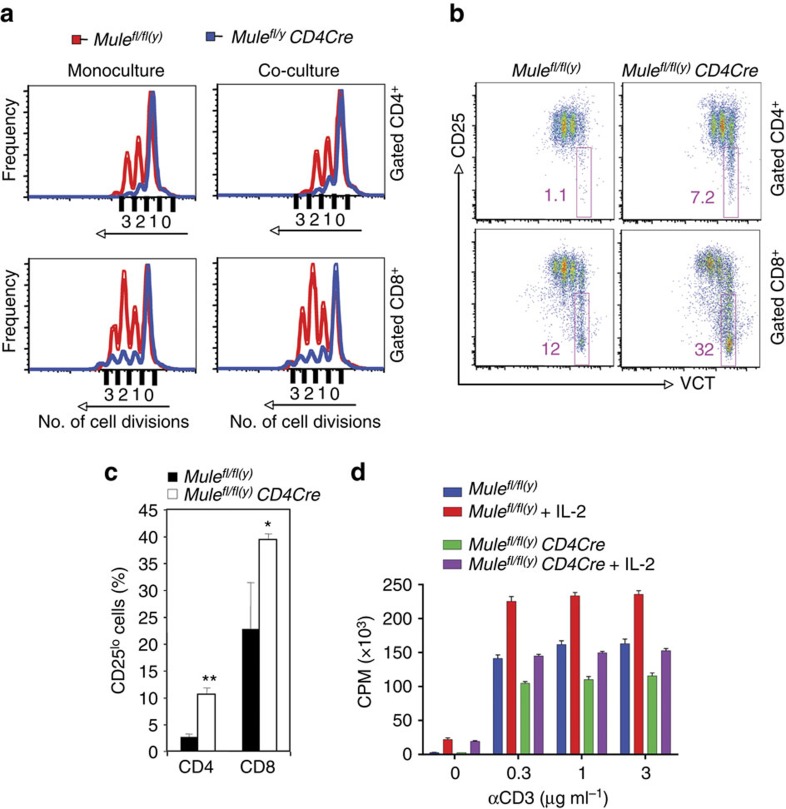
Intrinsic defect in the proliferation of *Mule*-deficient T cells. (**a**) VCT labelled T cells from control or TMKO mice were cultured alone (monoculture), or co-cultured with purified T cells from congenic SJL mice (CD45.1), and stimulated with anti-CD3/CD28 Abs for 48 h. Cell divisions tracked by VCT in gated CD4^+^ and CD8^+^ T cells were analysed by FCM, with each peak indicating one cell division. Results shown are one FCM profile representative of two independent experiments. (**b**) Purified T cells from control or TMKO mice were labelled with VCT and stimulated with anti-CD3/CD28 Abs for 48 h. Cell divisions tracked by VCT in gated CD4^+^ and CD8^+^ T cells, as well as surface CD25 levels as determined by anti-CD25 staining, were analysed by FCM. (**c**) Quantitation of the percentage of CD25^lo^ cells in **b**. CD4: *P*=0.0198; CD8: *P*=0.0317. Results are the mean±s.d. (*n*=3). (**d**) Quantitation of [^3^H]thymidine incorporation by control and TMKO splenocytes that were cultured for 48 h in medium containing the indicated doses of anti-CD3 in the absence or presence of exogenous IL-2 (50 U ml^−1^). Results are the mean±s.e.m. (*n*=3).

In theory, the impaired proliferation of TMKO T cells could be due to either an intrinsic activation defect or to an impaired response to cytokines and growth factors secreted during TCR-mediated activation. To address this question, we labelled T cells from WT CD45.1^+^ SJL B6 mice (control) and CD45.2^+^ TMKO T cells with VCT, mixed them at a 1:1 ratio, activated them with anti-CD3/28 Abs and determined their mitosis by FCM. The impaired cell division of TMKO CD4^+^ and CD8^+^ T cells cultured alone was maintained in the mixed cultures (Fig. 3a). Consistent with these data, *Mule*-deficient CD4^+^ and CD8^+^ T cells at 48 h post-stimulation showed a significantly higher fraction of CD25^lo^ cells in the non-dividing T-cell population than did control cultures (Fig. 3b,c), an impairment not rescued by the addition of exogenous IL-2 (Fig. 3d). Thus, the proliferation defect in TCR-stimulated TMKO T cells is cell-intrinsic rather than caused by a faulty response to growth factors/cytokines such as IL-2 secreted by activated T cells.

### Normal TCR signalling and apoptosis in *Mule*-deficient T cells

**Figure 4:**
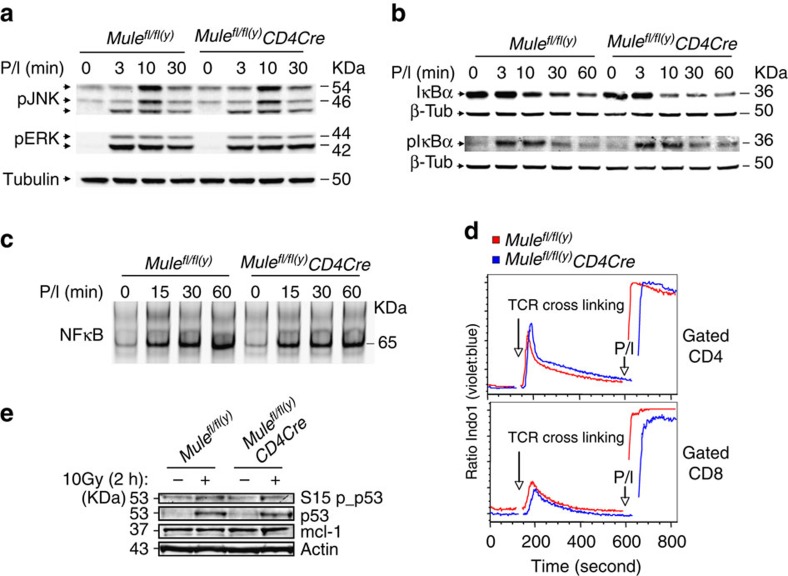
Normal TCR signalling in TMKO T cells. (**a**,**b**) IBs to detect (**a**) phospho-JNK1/2 (pJNK1/2) and phospho-Erk1/2 (pErk1/2), and (**b**) total IκBα and phospho-IκBα, in purified control and TMKO T cells that were stimulated with PMA/Ionophore (P/I) for the indicated times. Tubulin, loading control. (**c**) Gel mobility shift assay of nuclear extracts of the cells in **b** using radiolabelled probes detecting NFκB binding site sequences. (**d**) Ca2^+^ flux assay of control and TMKO LN cells that were preloaded with Indo-1 and incubated successively with hamster anti-mouse CD3 Ab, rabbit anti-hamster Ab and PMA/Iono. The Ca2^+^ response was measured based on the ratio of violet (Ca2^+^ bound) to blue (Ca2^+^ free) fluorescence in gated CD4^+^ and CD8^+^ T cells. (**e**) IB to detect phosphorylated p53, total p53 and Mcl-1 in purified control and TMKO T cells that were left untreated (−) or irradiated at 10 Gy (+). Cells were collected at 2 h post-treatment. Actin, loading control.

The elevation of Mule in response to TCR engagement and the requirement of Mule for TCR-mediated T-cell proliferation suggested that Mule might be involved in TCR-induced signal transduction. However, phosphorylation levels of JNK and ERK were comparable between PMA/Ionophore (PMA/Iono)-stimulated WT and TMKO T cells (Fig. 4a), as was IκBα phosphophorylation and degradation (Fig. 4b). Consistent with these findings, both NFκB DNA-binding activity as determined by EMSA (Fig. 4c) and Ca^2+^ flux (Fig. 4d) were comparable in PMA/Iono-stimulated WT and TMKO T cells. Thus, Mule is dispensable for normal activation of the MAP kinase and NFκB pathways as well as for calcium flux.

Because Mule's substrates p53 and Mcl-1 are involved in T-cell apoptosis^16^,^17^,^22^, we determined whether these molecules were altered in TMKO T cells. Levels of Mcl-1 protein as well as total p53 and phosphorylated p53 were comparable in WT and TMKO T cells treated with γ-irradiation (IR) (Fig. 4e). We then examined apoptosis in cultures of TMKO and WT thymocytes exposed to stauroporine, dexamethasone or IR and found comparable levels in all cases (Supplementary Fig. 2a). Because we previously showed that *Mule*-deficient B cells are resistant to DNA damage-induced apoptosis, and that the impaired B-cell development and homoeostasis in B-cell-specific *Mule* KO mice can be partially rescued by genetic ablation of *p53* (ref. 23), we bred TMKO mice with *p53*^*fl/fl*^ mice to generate T-cell-specific *Mule* plus *p53* double KO mice. Unlike in B cells, genetic ablation of p53 rescued neither the reduced CD4^+^ and CD8^+^ T-cell numbers in TMKO mice (Supplementary Fig. 2b) nor their proliferative defects (Supplementary Fig. 2c). Thus, loss of Mule-mediated ubiquitination and degradation of p53 and Mcl-1 is not responsible for the homoeostatic and proliferative defects of TMKO T cells.

### KLF4 is a novel Mule substrate

**Figure 5:**
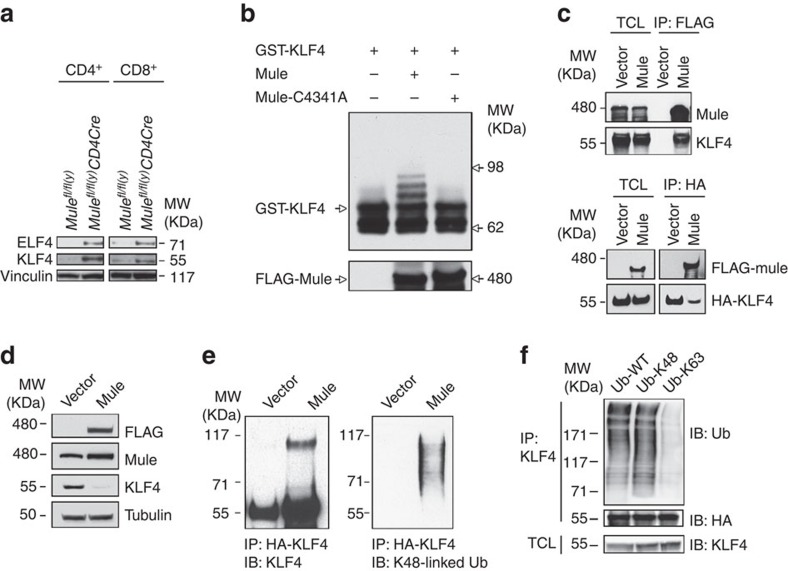
Identification of KLF4 as a novel Mule substrate. (**a**) IB to detect ELF4 and KLF4 in purified control and TMKO CD4^+^ and CD8^+^ T cells. (**b**) IB to detect ubiquitinated KLF4 after incubation of recombinant GST-KLF4 with ubiquitin, E1, HBCH5b (E2) and Mule (E3) or Mulec4341A mutant protein (lacks E3 ligase activity). Left lane, negative control; middle lane, assay with WT Mule; right lane, assay with Mulec4341A protein. (**c**) Upper panel: Lysates of 293T cells overexpressing vector control or Flag-Mule were transiently transfected with HA-KLF4 plasmid, IP'd with anti-Flag Ab and IB'd with anti-Mule and anti-KLF4 Abs. Lower panel: Lysates in the upper panel were IP'd with anti-HA Ab and IB'd with anti-Flag Ab to detect Mule, and with anti-HA Ab to detect KLF4. Total cell lysate was IB'd in parallel as a control. (**d**) IB to detect Flag, Mule and KLF4 in lysates of 293T cells overexpressing vector control or Flag-Mule. (**e**) 293T cells stably expressing either empty vector pCI-neo (left) or human Mule cDNA (right) were transiently transfected with HA-KLF4 plasmid and His-ubiquitin. Cell lysates were IP'd with anti-HA beads, and the beads were subjected to IB with either anti-KLF4 Ab (left) or anti-K48-linked Ub Ab (right). (**f**) 293T cells stably expressing Mule cDNA were transiently transfected with HA-Klf4 plasmid together with WT Ub, K48-only Ub or K63-only Ub. Cell lysates were IP'd with anti-HA beads, and the beads were IB'd with either anti-Ub or anti-HA Ab. Total cell lysate was IB'd with anti-KLF4 Ab. Results are representative of two to three independent experiments.

ELF4 and KLF4 play essential roles in TCR-mediated proliferation because these transcription factors regulate the cell cycle^9^,^10^. To determine whether Mule has effects on ELF4 and/or KLF4, we subjected purified WT and TMKO CD4^+^ and CD8^+^ T cells at steady-state to immunoblotting and detected substantial elevations of ELF4 and KLF4 proteins in TMKO T cells compared with the WT (Fig. 5a). This increase in ELF4 and KLF4 in *Mule*-deficient T cells suggested that these transcription factors might be Mule substrates. To test this hypothesis, we established an *in vitro* ubiquitination assay using Mule protein purified from Mule-overexpressing 293T cells. This *in vitro* assay revealed that KLF4 (but not ELF4) was indeed ubiquitinated by Mule (Fig. 5b). To confirm that this ubiquitination of KLF4 was due specifically to Mule's E3 ligase activity, we used site-directed mutagenesis to change the catalytic cysteine at Mule amino acid 4341 into an alanine (MuleC4341A), which totally abolishes Mule's E3 ligase activity^16^. KLF4 was not ubiquitinated when MuleC4341A was used in our *in vitro* assay instead of WT Mule protein (Fig. 5b). Furthermore, WT Mule and KLF4 bound to each other when tested by co-immunoprecipitation of Flag-tagged Mule or HA-tagged KLF4 (Fig. 5c). In contrast to the elevation of KLF4 in *Mule*-deficient T cells, KLF4 protein was decreased in Mule-overexpressing 293T cells (Fig. 5d). Notably, addition of the proteasome inhibitor MG132 restored KLF4 expression in Mule-overexpressing 293T cells to normal levels. Additional ubiquitination assays in Mule-overexpressing 293T cells confirmed that KLF4 protein immunoprecipitated using anti-HA Ab was indeed ubiquitinated, as determined by immunoblotting with either anti-KLF4 Ab or Ab recognizing Ub that could be attached only through K48 linkages (Ub-K48) (Fig. 5e). We then transfected our Mule-overexpressing 293T cells with WT Ub, Ub-48 or Ub that could be attached only through K63 linkages (Ub-K63). We found that KLF4 was ubiquitinated by Mule through a K48 linkage but not through a K63 linkage (Fig. 5f). Significantly, only substrates conjugated to Ub via K48 linkage are recognized and degraded by 26S proteasomes^28^. Our findings therefore identify KLF4 as a novel Mule substrate and indicate that its levels are controlled by K48-ubiquitination followed by degradation. This hypothesis is consistent with the observed KLF4 elevation in TMKO T cells and the KLF4 reduction in Mule-overexpressing 293T cells.

### Defective cell cycle regulation in *Mule*-deficient T cells

cMyc controls metabolic reprogramming during T-cell activation and proliferation^18^,^19^,^20^ and is a known Mule substrate^15^. Compared with cMyc in control T cells, cMyc protein was less highly elevated in *Mule*-deficient T cells stimulated by anti-CD3 Ab and its appearance was delayed (Supplementary Fig. 3a). However, when we examined cMyc's transcriptional activity by RT–PCR analysis, we detected normal mRNA levels of cMyc and its transcriptional targets in TMKO CD4^+^ T cells both at steady-state and at 3 h post-TCR stimulation (Supplementary Fig. 3b). Similar results were obtained for TMKO CD8^+^ T cells. Thus, the impaired activation of TMKO T cells is not due to effects on the cMyc pathway.

**Figure 6:**
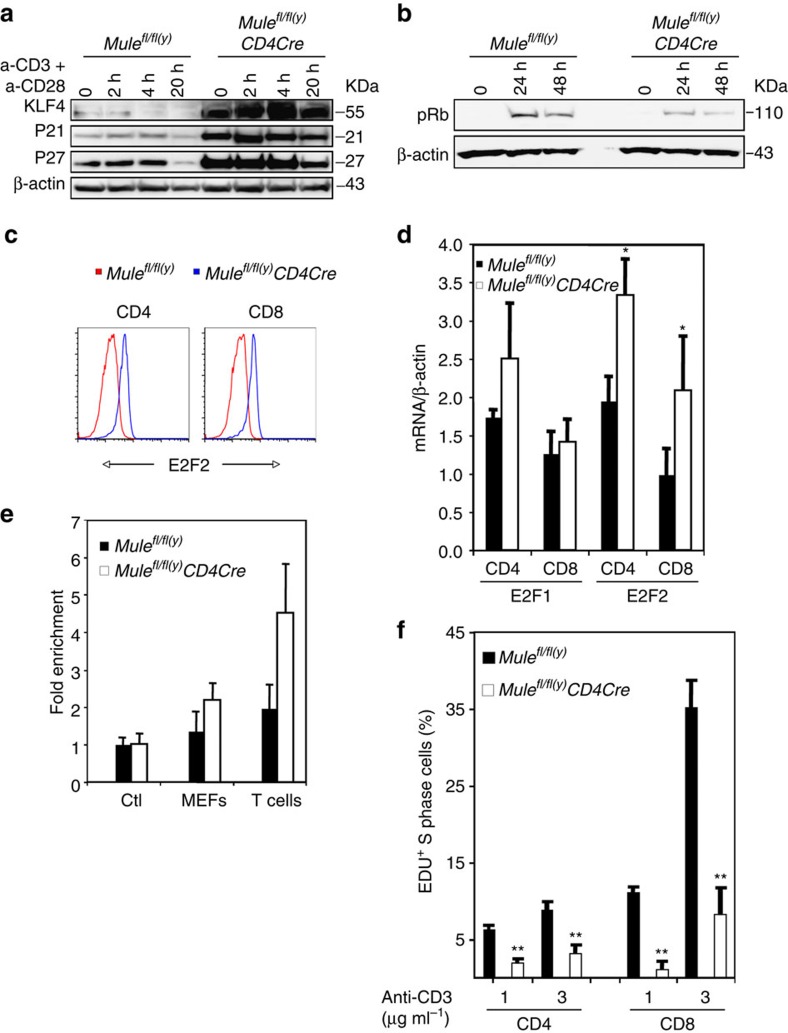
Activation of KLF4 in *Mule-*deficient T cells impairs cell cycle entry. (**a**) IB to detect KLF4, p21 and p27 proteins in purified control and TMKO T cells that were left untreated (0) or treated with anti-CD3/28 Abs for the indicated times. (**b**) IB to detect phospho-Rb in purified control and TMKO T cells that were left untreated (0) or treated with anti-CD3/28 Abs for the indicated times. (**c**) FCM analysis of E2F2 expression by gated purified control and TMKO CD4^+^ or CD8^+^ T cells that were fixed, permeabilized and subjected to intracellular staining with anti-E2F2 Ab. (**d**) Quantitation of RT–PCR analysis of E2F1 and E2F2 mRNA expression in purified, untreated control and TMKO CD4^+^ and CD8^+^ T cells. Data were normalized to β-actin mRNA and the relative change in gene expression was calculated using the comparative threshold cycle method (2ΔΔ^Ct^). Results are the mean±s.d. (*n*=3–4). (**e**) Quantitation of ChIP assays of the binding of KLF4 to the E2F2 enhancer in control (Ctl) and *Mule*-deficient MEFs, and in purified control and TMKO T cells. (**f**) Quantitation by EDU assay of the percentage of cycling cells in cultures of purified control and TMKO T cells that were treated for 24 h with anti-CD3/28 Abs at the indicated doses. Data are expressed as the percentage of S phase-positive T cells and are the mean±s.d. (*n*=3). *P<0.05; **P<0.005; Student's *t*-test; control versus TMKO. Results are representative of two to three independent experiments involving three to six mice per genotype.

We next turned to KLF4, since we now knew that this transcription factor, which controls T-cell proliferation through cell cycle regulators^9^,^10^, was a Mule substrate. We speculated that the defective proliferation of TCR-stimulated TMKO T cells might be caused by KLF4 that had accumulated due to the lack of Mule-mediated ubiquitination (followed by degradation) in these cells. Previous work has shown that KLF4 in WT T cells decreases to undetectable levels by 20 h post-stimulation with anti-CD3/CD28 Abs^9^. However, we found that KLF4 was significantly increased in unstimulated TMKO T cells and sustained at an elevated level for up to 20 h following TCR engagement (Fig. 6a and Supplementary Fig. 4c). Since KLF4 physically interacts with the promoters of the CDKI genes *p21* and *p27* and transactivates their expression^29^,^30^, we examined p21 and p27 levels in WT and TMKO T cells. While p21 and p27 proteins had declined in control T cells by 20 h post-stimulation, unstimulated TMKO T cells already contained abundant p21 and p27 proteins and maintained these high levels for at least 20 h post-stimulation (Fig. 6a). Because CDKIs inhibit Rb phosphorylation by binding to CDK complexes^31^,^32^,^33^, we investigated Rb activation in TMKO T cells. In support of our hypothesis, Rb was inefficiently phosphorylated in TMKO T cells, showing reductions to 35.3 and 26.3% of values in WT T cells at 24 and 48 h post-TCR engagement, respectively (Fig. 6b).

Another key transcriptional target of KLF4 is E2F2, which inhibits the entry of T cells into the cell cycle^13^,^14^. We found that the elevated KLF4 protein in TMKO CD4^+^ and CD8^+^ T cells was associated with increased E2F2 in these cells compared with controls (Fig. 6c). E2F2 mRNA levels were also increased by ∼1.7- and 2-fold in TMKO CD4^+^ and CD8^+^ T cells, respectively (Fig. 6d). In contrast, mRNA levels of the non-KLF4-driven transcriptional activator E2F1 were normal in TMKO T cells. Because KLF4 was shown to bind to the E2F2 enhancer in 3T3 L1 cells^34^, we used anti-KLF4 Ab to perform ChIP immunoprecipitation of WT and *Mule*-deficient mouse embryonic fibroblasts (MEFs), as well as WT and TMKO T cells. Consistent with the elevated KLF4 protein and E2F2 mRNA in TMKO T cells, RT–PCR analysis of the anti-KLF4 ChIP immunoprecipitation product showed enrichment of the E2F2 enhancer in *Mule*-deficient MEFs and TMKO T cells (Fig. 6e). Unlike other E2F family members, E2F2 is a transcriptional repressor that blocks mitosis such that *E2f2*-deficient T cells show enhanced S phase entry and hyperproliferate upon TCR stimulation^14^. Consistent with abnormal activation of the KLF4 pathway, cultures of TCR-stimulated TMKO CD4^+^ and CD8^+^ T cells showed significantly fewer S phase positive cells than controls (Fig. 6f).

Collectively, these results suggest that TCR-stimulated TMKO cells cannot ubiquitinate KLF4 in response to TCR engagement, and so this transcription factor is not degraded. The accumulating KLF4 protein drives high expression of p21, p27 and E2F2, which repress the entrance of T cells into the cell cycle. Thus, Mule controls T-cell proliferation through effects on KLF4 ubiquitination.

### Biased Th17 cell differentiation and severe EAE in TMKO mice

**Figure 7:**
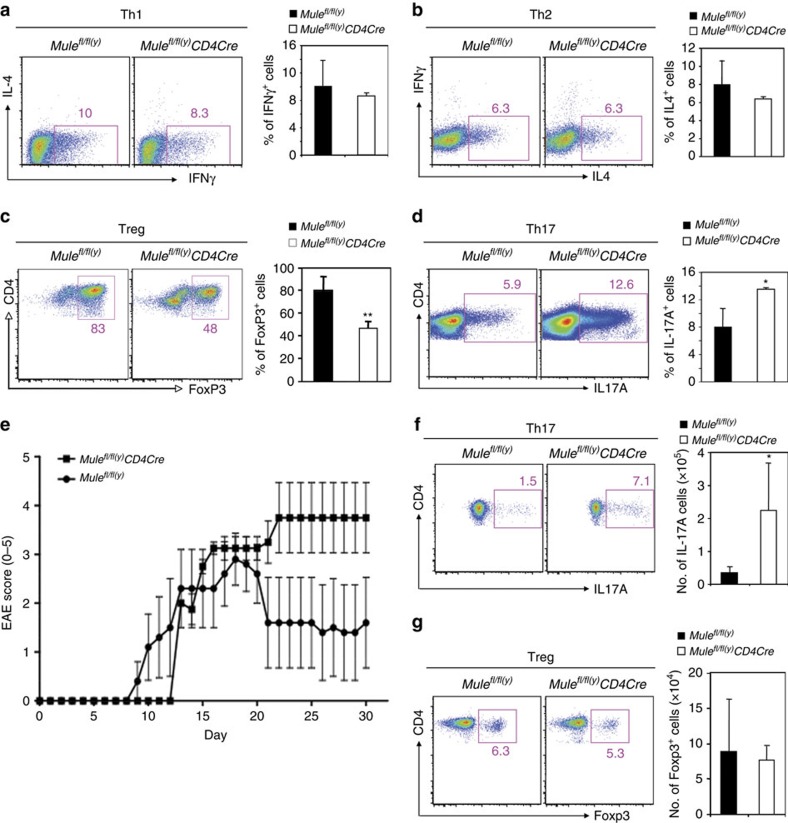
Abnormal Th17 differentiation of Mule-deficient CD4^+^ T cells and associated severe EAE development in TMKO mice. (**a**–**d**) Purified CD4^+^ T cells from control and TMKO mice (*n*=3–4/genotype) were cultured under Th1, Th2, Treg or Th17 polarizing conditions and subjected to intracellular immunostaining and FCM to detect production of the indicated cytokines/transcription factor. Left panels: Representative FCM plots showing percentages of cells secreting the indicated cytokines. Right panels: Quantitation of percentages of cells from the left panels secreting IFNγ (**a**), IL-4 (**b**), Foxp3 (**c**) or IL-17A (**d**). (c): *P*=0.0027; (d): *P*=0.043. Data are the mean±s.d. (*n*=3–4). (**e**) Time course of EAE induction in control and TMKO mice (*n*=4–5 mice/genotype) injected with MOG peptide. The severity of EAE development was scored using an established system^38^. Data are the mean EAE score±s.d. (*n*=4–5). (**f**,**g**) Total LN cells from control and TMKO mice 12 days post-EAE induction were cultured in the presence of PMA/Iono (**f**) or without culture (**g**) and subjected to intracellular immunostaining and FCM to detect production of IL-17A and Foxp3, respectively. Left panels: Representative FCM plots showing percentages of cells secreting the indicated cytokine/transcription factor. Right panels: Quantitation of cell numbers from the left panels secreting IL-17A (**f**) or Foxp3 (**g**). Data are the mean±s.d. (*n*=3).

To determine Mule's function in CD4^+^ Th cell differentiation, we induced purified WT and TMKO CD4^+^ T cells to undergo differentiation under Th1 or Th2 polarizing conditions. Despite their defect in TCR-stimulated proliferation, TMKO CD4^+^ T cells generated normal numbers of Th1 and Th2 cells (Fig. 7a,b). However, TMKO CD4^+^ T cells cultured under Treg polarizing conditions generated significantly fewer Foxp3^+^ Treg cells than did controls (Fig. 7c), whereas TMKO CD4^+^ T cells cultured under Th17 polarizing conditions generated markedly more IL-17-producing Th17 cells (Fig. 7d). Because Th17 cells are the pathological drivers of several human autoimmune and inflammatory disorders^35^,^36^, as well as murine EAE^37^, we injected control and TMKO mice with myelin oligodendrocyte glycoprotein (MOG) peptide emulsion plus pertussis toxin to induce EAE^38^ and found that EAE initiation in TMKO mice was slightly delayed (Fig. 7e). More strikingly, although control mice began to recover by day 20 post-MOG emulsion injection, TMKO mice continued to suffer from severe EAE (Fig. 7e). Consistent with their exacerbated EAE development, the draining inguinal LN in TMKO mice contained higher numbers of IL-17A-producing cells at 12 days post-EAE induction compared with EAE-induced controls (Fig. 7f). However, Treg cell numbers in draining LN were comparable in EAE-induced control and TMKO mice (Fig. 7g). Since *Mule*-deficient Treg cells showed normal capacity to suppress the proliferation of WT effector T cells *in vitro*, we speculate that the exacerbated EAE development in TMKO mice is not attributable to alterations in the Treg compartment. Rather, our data demonstrate that *Mule*-deficient Th17 cells are more pathogenic than WT Th17 cells in the EAE model. It is known that KLF4 binds to the *IL17a* promoter to positively regulate Th17 differentiation^11^,^12^, and that T-cell-specific *Klf4* KO mice show reduced Th17 cells and attenuated EAE development. We hypothesize that the KLF4 elevation in our TMKO T cells increases Th17 differentiation and thereby causes the exacerbated EAE development observed in TMKO mice.

### Impaired immune responses against LCMV in TMKO mice

**Figure 8:**
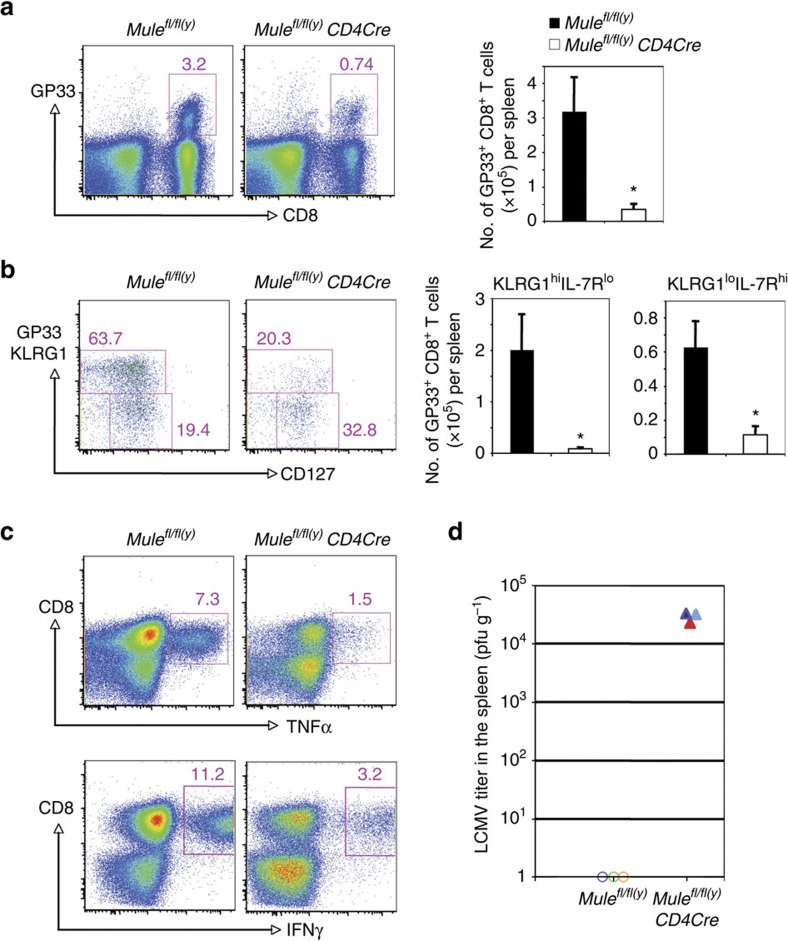
Impaired immune response to LCMV infection in TMKO mice. (**a**) Left panel: FCM analysis of GP_33–41_-specific CD8^+^ T cells in gated control and TMKO splenic lymphocytes. Right panel: Quantitation of absolute numbers of GP_33–41_-specific CD8^+^ T cells in the spleens of control and TMKO mice. Data are the mean±s.d. (*n*=3). (**b**) Left panel: FCM analysis of KLRG1 and CD127 expression by gated control and TMKO GP_33–41_-specific CD8^+^ T cells. Right panel: Quantitation of absolute numbers of KLRG1^hi^CD127^lo^ and KLRG1^hi^CD127^lo^ CD8^+^ T cells among the CD8^+^ T cells in the left panel. Data are the mean±s.d. (*n*=3). *P<0.05; Student's *t*-test, control versus TMKO. (**c**) FCM analysis of the production of the indicated cytokines by control and TMKO CD8^+^ T cells that were stimulated for 6 h with GP_33–41_ peptide and subjected to intracellular immunostaining. Data are representative of three mice/genotype. (**d**) Quantitation of splenic LCMV titers in control and TMKO mice at 7 days post-infection. Data points are values for individual mice. Results are representative of two independent experiments involving three mice per genotype.

To determine Mule's function in CD8^+^ effector T-cell differentiation, we infected WT and TMKO mice with LCMV and analysed CD8^+^ T-cell responses at 7 days post-infection. TMKO mice had significantly lower percentages and absolute numbers of splenic LCMV-specific GP_33–41_ CD8^+^ T cells compared with controls (Fig. 8a), suggesting impaired development of LCMV-specific CD8^+^ effector T cells. In infected WT mice, most GP_33–41_ CD8^+^ T cells were KLRG1^hi^CD127^lo^ short-lived effector cells, whereas this subset was markedly reduced in TMKO mice (Fig. 8b). After *in vitro* stimulation with GP_33–41_ peptide, the percentages of TNFα- and IFNγ-producing cells among TMKO T cells were much lower than in controls (Fig. 8c). Accordingly, unlike WT mice, TMKO mice failed to clear the virus from the spleen (Fig. 8d). Thus, loss of Mule in CD8^+^ T cells reduces LCMV-induced differentiation of CD8^+^ effector T cells, production of antiviral cytokines and virus clearance.

## Discussion

**Figure 9:**
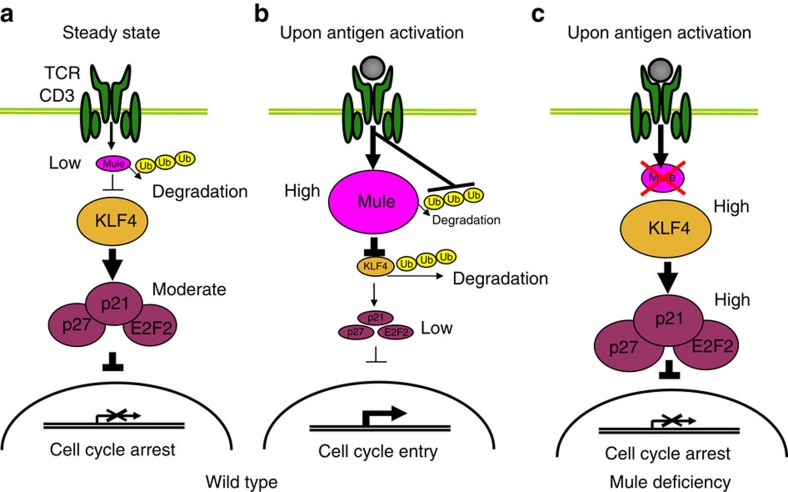
Model of mechanism by which Mule may control T-cell proliferation through ubiquitination and degradation of KLF4. (**a**) WT T cells at steady-state. In the absence of antigenic stimulation, Mule expression in T cells is low due to self-ubiquitination and degradation and its E3 ligase activity is insufficient to remove KLF4. KLF4 transactivates E2F2, which acts as a transcriptional repressor together with CDKI p21 and p27 to block entry into the cell cycle. (**b**) Antigen-stimulated WT T cells. In response to TCR engagement by antigen, Mule expression is rapidly increased and sustained due to inhibition of its self-ubiquitination and degradation. Mule ubiquitinates KLF4 and promotes its degradation such that insufficient KLF4 remains to successfully transactivate E2F2, p21 and p27. T cells can thus transcribe genes promoting cell cycle entry. (**c**) In antigen-stimulated *Mule*-deficient T cells, KLF4 cannot be degraded. The accumulating KLF4 protein transactivates E2F2, p21 and p27, leading to repressed expression of cell cycle genes. These T cells then fail to proliferate efficiently.

Our TMKO mice share many phenotypic defects, including impaired lymphocyte homoeostasis, activation and proliferation, with B-cell-specific *Mule* KO (BMKO) mice. However, while loss of p53 in BMKO mice rescues these defects^23^, p53 ablation in TMKO mice failed to restore T-cell activation and proliferation. Thus, Mule's main molecular effector in T cells differs from that in B cells. This effector is not cMyc, since the functions of this regulator and its transcriptional targets were not impaired in TMKO T cells. Instead, we identified the transcription factor KLF4 as a novel Mule substrate that is ubiquitinated by this E3 ligase and thus undergoes proteasomal degradation in T cells. Our results indicate that the elevated KLF4 in TMKO T cells blocks their TCR-mediated proliferation by increasing p21, p27 and E2F2, thereby inhibiting cell cycle entry. A model illustrating how Mule appears to control T-cell proliferation by orchestrating KLF4 degradation is shown in Fig. 9. In resting naive mature T cells (Fig. 9a), the low level of Mule protein present is not sufficient to facilitate the degradation of significant amounts of KLF4. Enough KLF4 is therefore present to activate expression of p21, p27 and E2F2, which transcriptionally repress genes promoting cell cycle entry^14^,^29^,^30^. As a result, in the absence of antigenic stimulation, T cells remain in the resting state. Given that Mule can ubiquitinate itself^39^, we speculate that Mule protein is constantly being degraded to maintain a low level in normal resting T cells. Upon TCR engagement by antigen and T-cell activation (Fig. 9b), Mule protein is rapidly elevated to a level that is sufficient to ubiquitinate most of the KLF4 present, driving KLF4's proteasomal degradation. We hypothesize that this TCR-induced increase in Mule is achieved by a blockade of self-ubiquitination imposed by factors modulated by TCR signalling. Upon the degradation of KLF4 mediated by elevated Mule, expression levels of p21, p27 and E2F2 are decreased, the inhibition mediated by these molecules is relieved, and the T cells can enter into S phase and proliferate. However, in *Mule*-deficient T cells (Fig. 9c), KLF4 cannot be ubiquitinated and efficiently degraded upon TCR stimulation, so that KLF4 accumulates and not only upregulates p21 and p27 but also maintains the E2F2 pathway in an activated state. Consequently, these *Mule*-deficient T cells cannot enter S phase and fail to proliferate vigorously in response to TCR engagement.

The above is a neat and tidy model, but in fact, the response of *Mule*-deficient T cells to TCR ligation was heterogeneous and some *Mule*-deficient T cells were able to proliferate. The normal Th1 and Th2 differentiation of *Mule*-deficient CD4^+^ T cells suggests that, at least *in vitro*, these cells can bypass the early proliferation defect through an unknown compensation mechanism and eventually undergo normal differentiation. In contrast, TMKO CD4^+^ T cells showed an abnormal increase in Th17 cell differentiation that was likely due to the elevated KLF4 levels present in proliferation-competent cells. This hypothesis is supported both by the reduced EAE development observed in T-cell-specific *Klf4* KO mice^11^,^12^, and by the severe EAE associated with increased Th17 cell differentiation displayed by our TMKO mice. Further investigation is required to understand the molecular mechanism(s) accounting for the heterogeneous response of TMKO CD4^+^ T cells to TCR engagement and their preference for Th17 differentiation.

We also showed that genetic ablation of Mule had a greater impact on the homoeostasis of CD8^+^ T cells than on that of CD4^+^ T cells. The defective cytokine production and impaired differentiation of TMKO CD8^+^ T cells in response to LCMV infection may be associated with their increased KLF4 and p27, as elevated p27 is known to prevent T-cell differentiation and to induce anergy^40^. While KLF4 was significantly elevated in TMKO T cells other transcription factors involved in CD8^+^ T-cell differentiation remain under investigation. Previous work has shown that Mcl-1 coordinates with Noxa to set the apoptotic threshold for selection of high-affinity T-cell clones during T-cell activation^41^. Given that Mcl-1 is a Mule substrate that accumulates in response to TCR engagement, it would be interesting to investigate if Mcl-1's function in affinity selection affects CD8^+^ T-cell differentiation.

It remains unclear how TCR engagement causes Mule to accumulate so quickly, and why this upregulated level of Mule is retained for at least 24 h post-TCR stimulation. We previously showed that Mule also accumulates rapidly in response to DNA damage^23^. Given that Mule levels rise significantly by 2 h post-stimulation, and that Mule self-ubiquitinates to control its stability and protein levels^39^, we speculate that TCR engagement may influence factors that are able to modulate Mule self-ubiquitination. For example, it has been shown that downregulation of the deubiquitination enzyme USP7S inhibits Mule self-ubiquitination and subsequent proteasomal degradation, thereby regulating Mule stability^42^. Further molecular investigations are needed to determine whether TCR stimulation downregulates USP7S to prevent Mule self-ubiquitination, which would promote Mule accumulation and drive KLF4 degradation.

It has long been known that ubiquitination mediated by E3 ligases regulates T-cell functions through the targeting of TCR proximal and downstream signalling components^5^,^43^. In our study, we have identified the transcription factor KLF4 as a novel target of Mule-mediated ubiquitination leading to proteasomal degradation. Mule has a crucial function in TCR-mediated proliferation because it controls levels of KLF4 and, by extension, levels of its transcriptional targets p21, p27 and E2F2. This novel regulatory mechanism sheds new light on our understanding of the control of TCR-mediated T-cell proliferation by identifying a mechanism that integrates ubiquitination with cell cycle entry. The importance of these multiple layers of E3 ligase-mediated ubiquitination is highlighted by the phenotype of Mule-deficient T cells, which exhibit defective homoeostasis and functional deficits. Thus, the E3 ubiquitin ligase Mule is a key player in the network of cell cycle regulators and transcription factors that control downstream elements of TCR signalling and ultimately autoimmune and antiviral immune responses.

## Methods

### Mice

*Mule*^*fl/fl(y)*^ mice generated by our laboratory previously^23^ were bred with *CD4Cre*^24^ or *CD2Cre*^25^ Tg mice imported from Jackson laboratory to generate *Mule*^*fl/fl(y)*^*CD4Cre* and *Mule*^*fl/fl(y)*^*CD2Cre* mice, respectively. These animals (collectively, TMKO mice) were backcrossed for 6–10 generations to C57BL/6. Because Cre expression can be toxic^44^, we included *Mule*^*fl/+*^*CD4Cre* and *Mule*^*fl/+*^*CD2Cre* mice in all initial analyses of the corresponding mutants. These control mice were phenotypically indistinguishable from the control *Mule*^*fl/fl(y)*^ animals used in this study, as judged by cellular composition and size of spleen and LN. Both male and female mice used in the same ratio for experiments were at the age of 6–20 weeks unless otherwise specified. All animal experiments were approved by the University Health Network Animal Care Committee.

### Flow cytometry

Lymphoid cells prepared from spleen, LN or thymus of *Mule*^*fl/fl(y)*^ and *Mule*^*fl/fl(y)*^*CD4Cre* animals were treated to lyse red blood cells. About 1–2 million cells were preincubated with anti-CD16/CD32 Ab (2.4G2, 1:100) to block FcR for 15 min at 4 °C and immunostained with different combinations of fluorochrome-conjugated Abs recognizing the following: CD3 (145-2C-11, 1:50), CD4 (GK1.5, 1:100), CD5 (53-7.3, 1:50), CD8 (53-6.7, 1:50), CD25 (PC61, 1:50), CD44 (IM7, 1:100), CD62L (MEL-14, 1:50) or CD69 (H1.2F3, 1:50) (all from BioLegend, BD Biosciences or eBioscience).

For BrdU staining, *Mule*^*fl/fl(y*^ and *Mule*^*fl/fl(y)*^*CD4Cre* mice were supplied with BrdU-containing drinking water (1 mg ml^−1^) for 3 days. Single cell suspensions of total LN cells were immunostained with anti-CD4 (GK1.5, 1:50) and anti-CD8 (53-6.7, 1:50) Abs. BrdU incorporation by each subset was detected by flow cytometry using the BrdU-Flow kit according to the manufacturer's instructions (BD).

Protein detection by intracellular staining was performed as described previously^45^. Briefly, cells were fixed with 1.6% paraformaldehyde and incubated for 30 min at room temperature (RT). After one wash in phosphate-buffered saline (PBS), ice-cold methanol (100%) was added dropwise and cells were incubated on ice for 30 min. Washed cells were incubated for 30 min with Abs recognizing CD3 (145-2C-11, 1:50), CD4 (GK1.5, 1:50), CD8 (53-6.7, 1:50) or E2F2 (Abcam). FCM data were acquired using a BD FCMCanto flow cytometer and analysed with FlowJo software (Tree Star Inc.).

### Violet cell tracker labelling

Purified T cells (1 × 10^6^ ml^−1^) from *Mule*^*fl/fl(y)*^, *Mule*^*fl/fl(y)*^*CD4Cre* or congenic SJL mice (CD45.1) mice were incubated with 2.5 μM CellTracker Violet (VCT; Life Technologies) in phosphate-buffered saline (PBS) for 10 min at 37 °C. Culture medium containing 10% foetal calf serum (FCS) was added to 5 × the original staining volume and incubation was continued for 5 min at RT. Cells were washed and resuspended at a density of 1 × 10^6^ cells ml^−1^ in pre-warmed RPMI-1640 medium containing 10% FCS. Cells were either seeded onto plates coated with anti-CD3 plus anti-CD28 Abs, or mixed with lethally irradiated CD45.1^+^ splenocytes loaded with GP_33–41_ peptide, and cultured for 2–3 days. The proliferation capacity of the stimulated T cells was measured by FCM as determined by a decrease in VCT fluorescence intensity.

### Ca^2+^ flux

LN cells from *Mule*^*fl/fl(y*^ and *Mule*^*fl/fl(y)*^*CD4Cre* mice were immunostained with anti-CD4 and anti-CD8 Abs as described above. Stained cells (1 × 10^7^) were incubated at 37 °C for 45 min with 5 μg ml^−1^ Indo-1 AM (Invitrogen) in RPMI-1640 medium supplemented with 10% FCS. Indo-loaded cells were resuspended at 5 × 10^6^ ml^−1^ and incubated with anti-CD3 (10 μg ml^−1^) on ice for 20 min. Cell aliquots (500 μl) were warmed to 37 °C for 5 min and a baseline reading was acquired for 3 min. Ca^2+^ flux induction was triggered by the addition of 2.4 μg ml^−1^ rabbit anti-hamster Ab (Jackson ImmunoResearch Laboratories, Inc.) followed 7 min later by addition of 100 ng ml^−1^ PMA plus 1 ng ml^−1^ Ca^2+^ ionophore A23187 (Sigma). Data collection was continued for another 3 min. The Ca^2+^ response was measured based on the ratio of violet (Ca^2+^ bound) to blue (Ca^2+^ free) fluorescence as detected by FCM (LSRII; BD).

### Mule-overexpressing and MuleC4341A-overexpressing 293T cell lines

We transfected 293T cells (from ATCC) with 3 × Flag-pCI-neo plasmid expressing either human Mule or MuleC4341A (see below) cDNA. Neomycin-resistant clones were selected by culture in G418 (700 μg ml^−1^) for 7–10 days. Single cells were sorted into 96-well plates and individual clones allowed to grow into cell lines. Clones expressing abundant Mule or MuleC4341 protein were identified by immunoblotting analysis. Selected Mule-overexpressing or MuleC4341A-overexpressing cells were expanded in number and used to produce the Mule or MuleC4341 proteins, respectively, which were employed for *in vitro* ubiquitination assays (see below).

### Immunoblotting and immunoprecipitation

For immunoblotting, every 1 × 10^7^ cells were lysed in 100 μl of 0.5% NP-40 lysis buffer (20 mM Tris-HCl pH 8, 137 mM NaCl, 10% glycerol, 0.5% NP-40, 2.5 mM EDTA), or RIPA buffer (50 mM Tris-HCl pH 8, 150 mM NaCl, 0.5% NP-40, 0.5% sodium deoxycholate, 0.1% SDS), with protease inhibitor and phosphatase inhibitor (Roche) freshly added. Lysates were vortexed for 15 s, incubated on ice for 30 min and cleared by centrifugation. Protein concentrations in lysates were determined using the BCA protein assay (Thermo Scientific). Cell lysates were loaded onto 4–12% bis-Tris gels to detect proteins below 200 kDa or 3–8% Tris-acetate gels to detect proteins above 200 kDa. For immoprecipitations, lysates of 293T cells were incubated with anti-Flag (M2; Sigma) or anti-HA (HA-7; Sigma) Ab-coated Protein G-Sepharose beads at 4 °C for 2 h to overnight. Immunocomplexes were washed 5 × with lysis bufunfer and subjected to SDS/PAGE. Fractioned proteins were transferred to a nitrocellulose membrane by i-Blot according to the manufacturer's instructions (Invitrogen) and immunoblotted using Abs recognizing the following: ubiquitin (FK2; Enzo Life Sciences; 1:1,000); Lys-specific ubiquitin (Apu2; Millipore; 1:1,000); KLF4 (GeneTex; 1:1,000 ); Mule (Bethyl Laboratories Inc.; 1:1,000); p21 (Santa Cruz; 1:1,000); p27 (BD Biosciences; 1:1,000); p53 (Santa Cruz; 1:1,000); Ser18-p53 (R&D; 1:1,000); or phospho-Erk1/2, phospho-JNK1/2, IκBα, phospho-IκBα, p65 or phospho-IKKα/β (all from Cell Signaling; 1:1,000). Infrared dye-labelled secondary Abs (anti-rabbit Alexa 680, Invitrogen; 1:10,000 and anti-mouse IR800; 1:20,000, LICOR) were visualized with an Odyssey scanner (LICOR). The whole gel images of western blots are presented in Supplementary Figs 4–6.

### *In vitro* assay of Mule-mediated KLF4 ubiquitination

293T cells overexpressing Mule or MuleC4341A (see below) were grown to near-confluence. Lysates were prepared using standard methods and incubated with Anti-Flag M2 affinity gel beads (Sigma) at 4 °C with rotation for 2 h. Flag-Mule or Flag-MuleC4341A was eluted by adding 3 × Flag peptide (Sigma) at a final concentration of 100 μg ml^−1^ in 1 × ubiquitination buffer (50 mM Tris pH 7.4, 2 mM ATP, 5 mM MgCl_2_ and fresh 2 mM DTT) to the washed beads. Tubes were rotated at 4 °C for 5–10 min. For the KLF4 ubiquitination assay, 0.1 ng human E1, 0.4 ng Ubc5/7 E2, 3 μl of eluted Mule or MuleC4341 protein, 2 mM ubiquitin and 2 mM fresh ATP were mixed in ubiquitination buffer to a final volume of 20 μl. The reaction mixtures were incubated in a PCR machine at 30 °C for 90 min, when 5 μl SDS–PAGE buffer (5 × ) was added to stop the reaction. Ubiquitinated proteins were separated by 4–12% SDS–PAGE and detected by immunoblotting with anti-ubiquitin (FK2; Enzo Life Science) or anti-KLF4 (GeneTex) Ab.

### *In vitro* assay of Mule-mediated KLF4 ubiquitination

Mule-overexpressing 293T cells and control 293T cells were transiently transfected with HA-KLF4 plasmid and His-ubiquitin, and cultured for 24 h. For ubiquitin linkage experiments, these cells were transiently transfected with HA-KLF4 plasmid together with either WT ubiquitin, K48-only ubiquitin or K63-only ubiquitin (Addgene plasmids #17605 and 17606). Transfected cells were treated with MG132 (10 μM) for 6 h to prevent any proteasome-mediated degradation of ubiquitinated proteins. Cell lysates were immunoprecipitated with anti-HA beads. The beads were subjected to immunoblotting with anti-KLF4, anti-K48-linked Ub, anti-ubiquitin or anti-HA Abs. Total cell lysates were immunoblotted with anti-KLF4 Ab.

### [^3^H]-Thymidine incorporation

Purified T cells from *Mule*^*fl/fl(y*^ and *Mule*^*fl/fl(y)*^*CD4Cre* mice were seeded in triplicate in 96-well U-bottom plates at 1 × 10^5^ cells per 200 μl in RPMI-1640 containing 10% FCS. Cells were left untreated, or stimulated with various amounts of anti-CD3 Ab (#2C-11–45, BD Biosciences) or with anti-CD3 plus 1 μg ml^−1^ of anti-CD28 (#37.51, BD Biosciences). At 16 h post-seeding with stimuli, [^3^H]-thymidine (1 μCi) was added to each well and cells were cultured for another 8 h to determine proliferation at 24 h. [^3^H]-thymidine uptake was assessed using a liquid scintillation β-counter (TopCount reader).

### LCMV infection

Mice with a genotype of *Mule*^*fl/fl(y*^ and *Mule*^*fl/fl(y)*^*CD4Cre* mice were infected with 2000 pfu LCMV (Armstrong) by IV injection and bled 7 days post-infection. The proportions of CD8^+^ T cells that were specific for LCMV GP_33–41_ among lymphocytes in peripheral blood and spleen were assayed using either PE- or APC-conjugated H-2D^b^/ GP_33–41_ (KAVYNFATM) at 1:50 dilution at 4 °C for 1 h followed by FCM^46^.

### Gel mobility shift assay

Nuclear extracts of purified T cells from *Mule*^*fl/fl(y*^ and *Mule*^*fl/fl(y)*^*CD4Cre* mice were prepared according to a standard protocol. Extract (4 μg) was mixed with 2 μg poly(dI-dC) (Pharmacia) plus infrared dye end-labelled DNA oligonucleotides (LICOR) specific for NFκB, and incubated for 30 min at RT according to the manufacturer's protocol. Complexes were visualized using an Odyssey scanner (LICOR).

### Generation of MuleC4341A by site-directed mutagenesis

Full-length human Mule cDNA (4374 amino acids) was the kind gift of Dr Qing Zhong (UT Southwestern Medical Center). Using the Quick Change Mutagenesis II ^TM system (Stratagene), a silent SacII site was introduced into this cDNA in the region spanning amino acids 3881 to 3883 by changing bases cag cct gct to ca*a* cc*c* gc*g*. Primers used for creation of the silent SacII site were: 5′-CGG TGC TAG TGC TAC AAC CCG CGG TCG AGG CCT TCT TTC TGG-3′ and 5′-CCA GAA AGA AGG CCT CGA CCG CGG GTT GTA GCA CTA GCA CCG-3′. For mutation of the active cysteine regulating Mule E3 ligase activity^16^, a C to A mutation of amino acid 4341 was introduced by changing bases aca tgt to ac*c* *gc*t via site-directed mutagenesis. Primers used were: 5′-CCT GCC TTC AGC TCA CAC CGC TTT TAA TCA GCT GGA TCT G-3′ and 5′-CAG ATC CAG CTG ATT AAA AGC GGT GTG AGC TGA AGG CAG G-3′. All PCR-generated mutagenized fragments were completely sequenced to verify their sequences. Following mutagenesis and sequencing, the∼3 kb Nhe1/Not1 C-terminal fragment, containing both the silent SacII site and the C4341A mutation, was ligated to an∼3.5 kb ApaI/NheI hMule middle fragment and an ∼7.9 kb SalI/ApaI N-terminal fragment of hMule. This reconstructed cDNA was subcloned into a pCI_Neo plasmid backbone with an N-terminal 3 × Flag tag and 12 × His tag to generate the ∼19 kb full-length pCI-Neo-12His-3 × Flag MuleC4341A.

### Real-time PCR

RNA extracted from purified CD4^+^ or CD8^+^ T cells from *Mule*^*fl/fl(y*^ and *Mule*^*fl/fl(y)*^*CD4Cre* mice was reverse-transcribed as per the manufacturer's protocol (Invitrogen). The resulting cDNAs served as templates for real-time PCR reactions using Power SYBR-Green PCR Master Mix (Applied Biosystems) and an ABI 7700HT Fast Real-Time PCR System (Applied Biosystems). Data were analysed using SDS software provided by Applied Biosystems.

The mRNA expression of E2F2 was analysed using the following primer sequences: e2f1 forward, 5′-gacatcaccaatgtcctggag-3′; e2f1 reverse, 5′-cttcaagccgcttaccaatc-3′; e2f2 forward, 5′-tggagggtatccagctcatc-3′; e2f2 reverse, 5′-agctggtccaaggtctgct-3′. Each sample was assessed in triplicate. Relative mRNA levels were normalized to the housekeeping gene *S18* and calculated using the comparative threshold cycle method (2-ΔΔ^Ct^).

The mRNA expression of cMyc target genes was determined by RT–PCR using custom RT^2^ Profiler PCR Arrays from Qiagen. The relative change in gene expression was calculated by Qiagen's Data Analysis Centre, which set the value of the untreated control group to 1. Relative mRNA levels were normalized to the housekeeping gene β-actin.

### Cytokine and transcription factor detection

For analyses of cytokine profiles in mice with genotype of *Mule*^*fl/fl(y*^ and *Mule*^*fl/fl(y)*^*CD4Cre* mice at 12 days post-EAE induction, single cell suspensions prepared from draining LN (inguinal) were stimulated for 5 h with 50 nM PMA plus 750 nM Iono (Sigma-Aldrich) in the presence of Golgi Plug (1: 1,000; BD Biosciences)^47^. For Th1 and Th2 differentiation, purified naive CD4^+^ T cells (5 × 10^6^ ml^−1^) from spleen and/or LNs were stimulated with anti-CD3 (1 μg ml^−1^; 145-2C11) plus 10 μg ml^−1^ anti-IL-4 (11B11) for Th1 differentiation, or 10 μg ml^−1^ anti-IFNγ (XMG1.2) for Th2 differentiation. After overnight culture, Th1 cultures received 50 U ml^−1^ recombinant murine IL-2, whereas Th2 cultures received 50 U ml^−1^ murine IL-2 plus 500 U ml^−1^ murine IL-4. After 3–5 days culture, Th1 and Th2 cells were restimulated with anti-CD3 for 6 h in the presence of GolgiPlug (BD Biosciences). The proportions of Th1 cells secreting IFNγ and Th2 cells secreting IL-4 were determined by intracellular anti-IL4 (11B11, BD Biosciences, 1:50) and anti- IFNγ (XMG1.2, Biolegend, 1:50) antibody staining. For Th17 cell differentiation, purified naïve CD4^+^ T cells (2–10 × 10^6^ ml^−1^) were cultured at a ratio of 1:1 with 3,000-rad irradiated C57BL/6 splenocytes in the presence of 20 ng ml^−1^ IL-6 (Peprotech), 10 ng ml^−1^ IL-23 (R&D Systems), 3 ng ml^−1^ TGFβ (R&D Systems), 10 μg ml^−1^ anti-IL-4 (11B11) and 10 μg ml^−1^ anti-IFNγ (XMG1.2). On day 2, cultures received 50 U ml^−1^ murine IL-2 (Biosource). On day 5, cells were stimulated with anti-CD3 for 4 h in the presence of GolgiPlug. The proportion of IL-17-producing cells was determined by intracellular cytokine staining using anti-IL-17 antibody (BD Biosciences, TC11-18H10). For Treg cell differentiation, purified naïve CD4^+^ T cells (5 × 10^6^ ml^−1^) were stimulated with anti-CD3 (1 μg ml^−1^; 145-2C11) plus 4 ng ml^−1^ TGF-β, 50 U ml^−1^ IL-2, 5 μg ml^−1^ anti-IFN-γ (XMG1.2) and 1 μg ml^−1^ anti-CD28 (37.51). After 3 days culture, cells were stimulated for 5 h with 50 nM PMA plus 750 nM Iono (Sigma-Aldrich) in the presence of Golgi Plug (1: 1,000; BD Biosciences). The stimulated cells were incubated with anti-CD16/CD32 Ab (2.4G2, 1:100) to block FcR and then fixed and permeabilized using the Foxp3 detection kit (eBioscience) according to the manufacturer's instructions, followed by FCM.

### Chromatin immunoprecipitation (ChIP) assay

Crosslinked MEFs or purified T cells from *Mule*^*fl/fl(y*^ and *Mule*^*fl/fl(y)*^*CD4Cre* mice were lysed in ChIP lysis buffer. Samples were sonicated and centrifuged for 10 min at 10,000*g*. Supernatants were incubated at 4 °C overnight with anti-KLF4 Ab (sc-20691; Santa Cruz) plus Protein G-Sepharose beads in a 100 μl volume. Chromatin complexes were washed, eluted and reverse-crosslinked. Purified precipitated DNA was used for RT–PCR as described above. Primer sequences were: control-forward: 5′-CCAATACAGATGGGGAGGCT-3′, control-reverse: 5′-CCCGTTAATGCCAGAAAAGG-3′; e2f2-enhancer-forward: 5′-AGACTCCCTAAAAGCCTGCC-3′, e2f2-enhancer-reverse: 5-AGCAGCTCCAGGTCACAC-3′.

### Statistical analyses

The Student's *t*-test was employed for statistical analyses (one-sided and unpaired). Analyses were performed by manuscript authors who were blinded to the experimental group designations. Values are expressed as the mean±s.d. and each experiment was repeated at least twice. For statistical significance: *P<0.05; **P<0.005; ^***^P<0.0005.

### Data availability

The authors declare that the data supporting the findings of this study are available within the article and its Supplementary Information Files, or from the corresponding authors on a reasonable request.

## Additional information

**How to cite this article:** Hao, Z. *et al*. K48-linked KLF4 ubiquitination by E3 ligase Mule controls T-cell proliferation and cell cycle progression. *Nat. Commun.* **8,** 14003 doi: 10.1038/ncomms14003 (2017).

**Publisher's note:** Springer Nature remains neutral with regard to jurisdictional claims in published maps and institutional affiliations.

## Supplementary Material

Supplementary Information Supplementary Figures 1-6.
