# Supplementary material for: K48-linked KLF4 ubiquitination by E3 ligase Mule controls T-cell proliferation and cell cycle progression
Source: Nat Commun. 2017 Jan 13;8:14003. doi: 10.1038/ncomms14003 (PMC5241832; doi:10.1038/ncomms14003)
Supplement: Supplementary Information — Supplementary Figures 1-6. [file ncomms14003-s1.pdf]

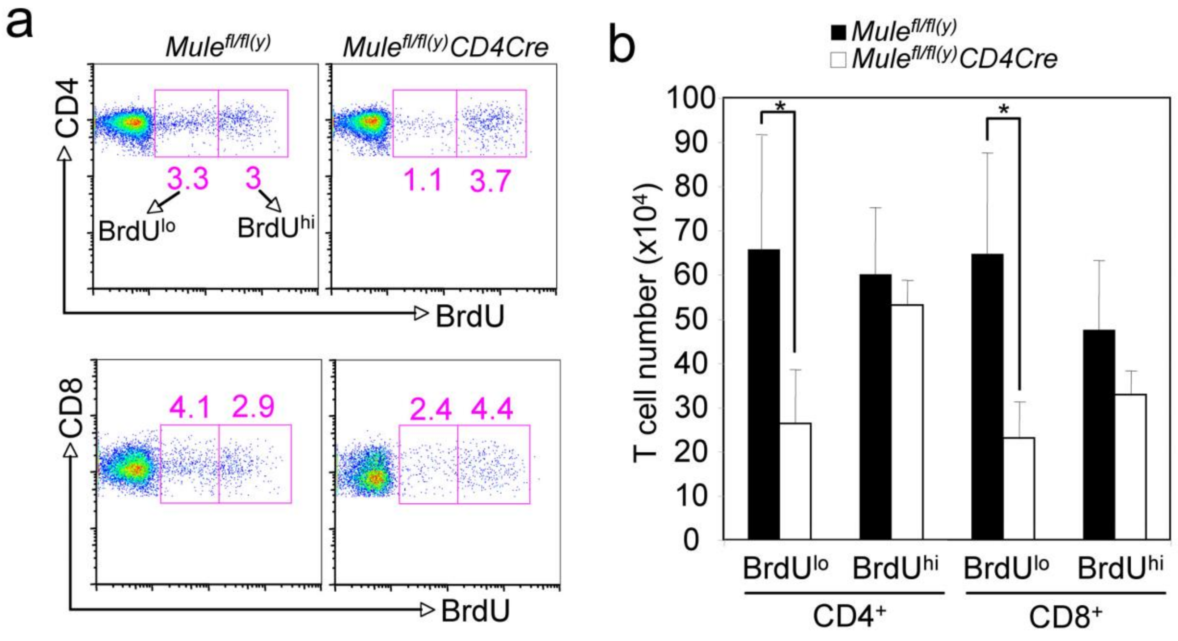

Supplementary Figure 1

Supplementary Figure 1. Reduction of CD4$^+$BrdU^{lo} and CD8$^+$BrdU^{lo} T cells in TMKO mice. (**a**) Control and TMKO mice were supplied with BrdU-containing drinking water for 3 days. Total lymph node (LN) T cells were stained with anti-BrdU and anti-CD4 and -CD8 Abs, and analyzed by flow cytometry. Numbers are the percentage of BrdU^{lo} and BrdU^{hi} T cells among gated CD4$^+$ and CD8$^+$ T cells, as indicated. (**b**) Quantitation of numbers of CD4$^+$ and CD8$^+$ BrdU^{lo} and BrdU^{hi} T cells among the cells in (a). Results are the mean ± SD (n=3);_*p<0.5. P values were calculated with one-sided Student's t-test.

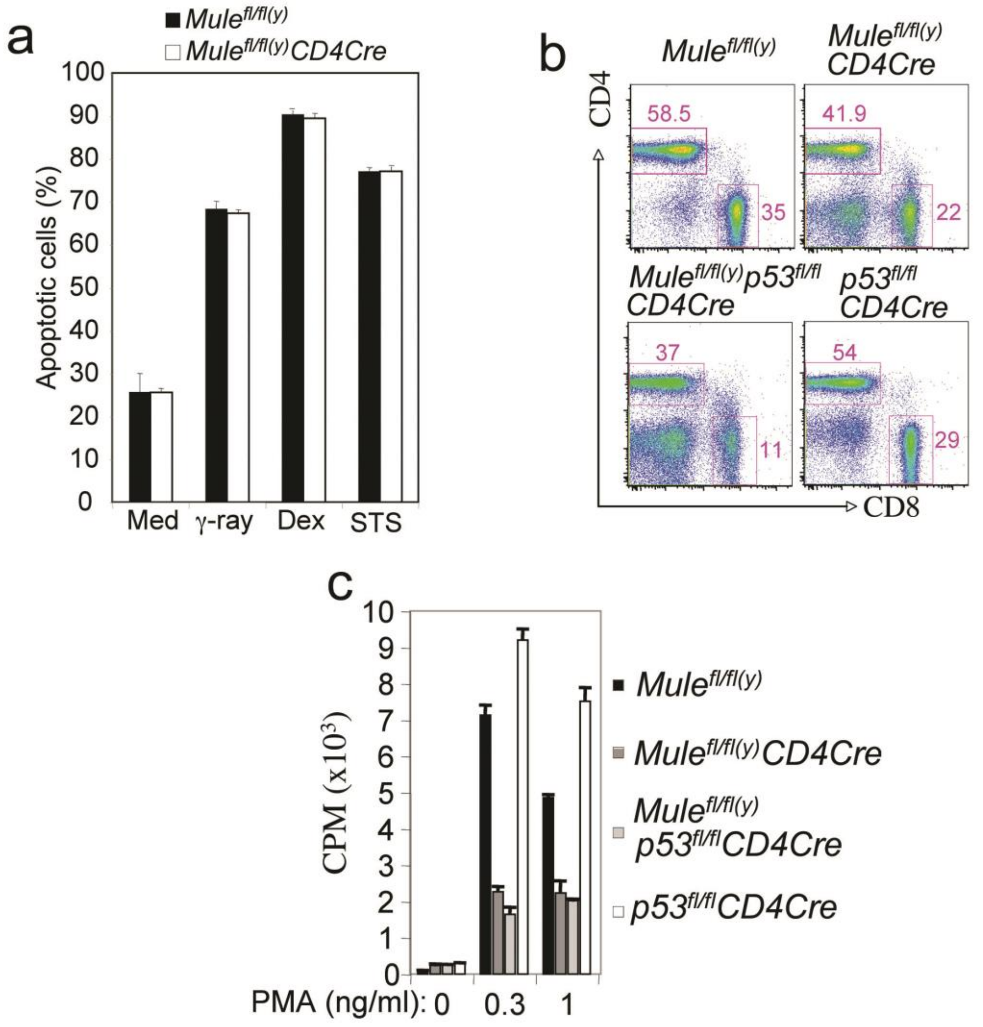

Supplementary Figure 2

Supplementary Figure 2. Normal apoptosis of *Mule*-deficient thymocytes and p53-independence of impaired TMKO T cell homeostasis. (**a**) Thymocytes from control and TMKO mice were left untreated in medium (Med), or treated for 20–24h with γ-irradiation (100 rads), dexamethasone (Dex, 10nM) or staurosporine (STS, 0.1 μM). Cells were stained with Annexin V/7-AAD and viability (AnnV$^+$7-AAD$^+$ cells) was determined by FCM analysis. Results are the mean percentage ± SD (n=3); P>0.5._P values were calculated with one-sided Student's t-test. (**b**) FCM analysis of CD4 vs. CD8 staining of LN cells from control, TMKO, T cell-specific *Mule/p53* double KO, and T cell-specific *p53* KO mice. Numbers are the percentages of CD4$^+$ and CD8$^+$ T cells

among gated lymphocytes. (**c**) Proliferation as determined by [^{3}H]thymidine incorporation of purified T cells that were isolated from the mice in (**b**) and cultured for 24h in medium alone (0), or medium containing PMA (0.3 ng/ml or 1ng/ml) plus ionophore (100ng/ml). Results are the mean ± SD of triplicate samples and are representative of 2 (a, b) or one (c) independent experiments.

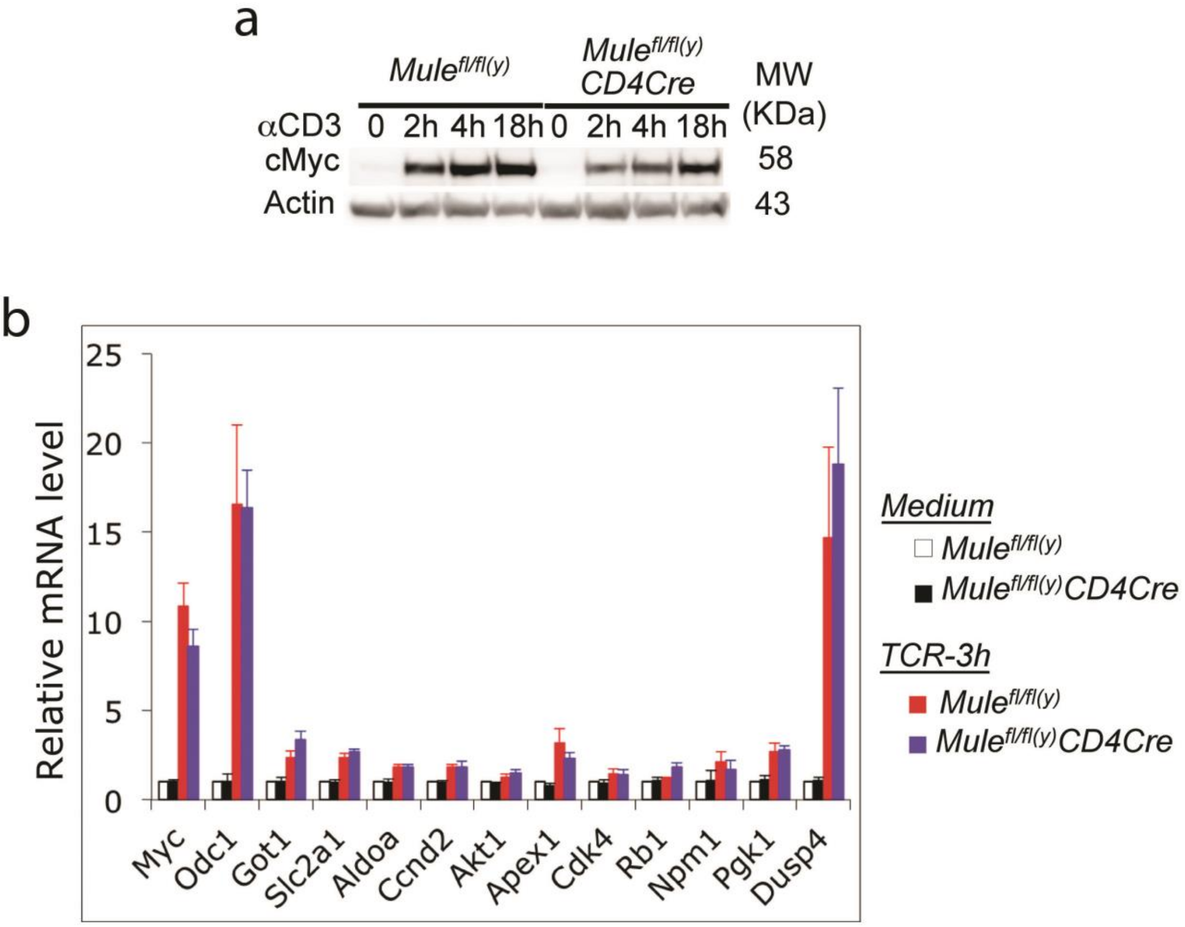

Supplementary Figure 3

Supplementary Figure 3. Normal expression of cMyc transcriptional targets in *Mule*-deficient $CD4^+$ T cells.

(a) Immunoblot to detect cMyc in purified control and TMKO T cells that were cultured in medium

alone (0) or in the presence of anti-CD3 (3μg/ml) plus anti-CD28 (1μg/ml) Abs for the indicated times. Actin, loading control. (b) Purified $CD4^+$ T cells from control and TMKO mice (n=3-5/group) were cultured in medium alone or in the presence of anti-CD3 (3μg/ml) plus anti-CD28 (1μg/ml) Abs (TCR-3h) for 3h. The mRNA levels of the indicated cMyc target genes were determined by quantitative RT-PCR using custom RT^2 Profiler PCR Arrays from Qiagen. The relative change in gene expression was calculated by Qiagen's Data Analysis Centre using the untreated control group set to 1. Results are the mean ± SD after normalization to β-actin mRNA.

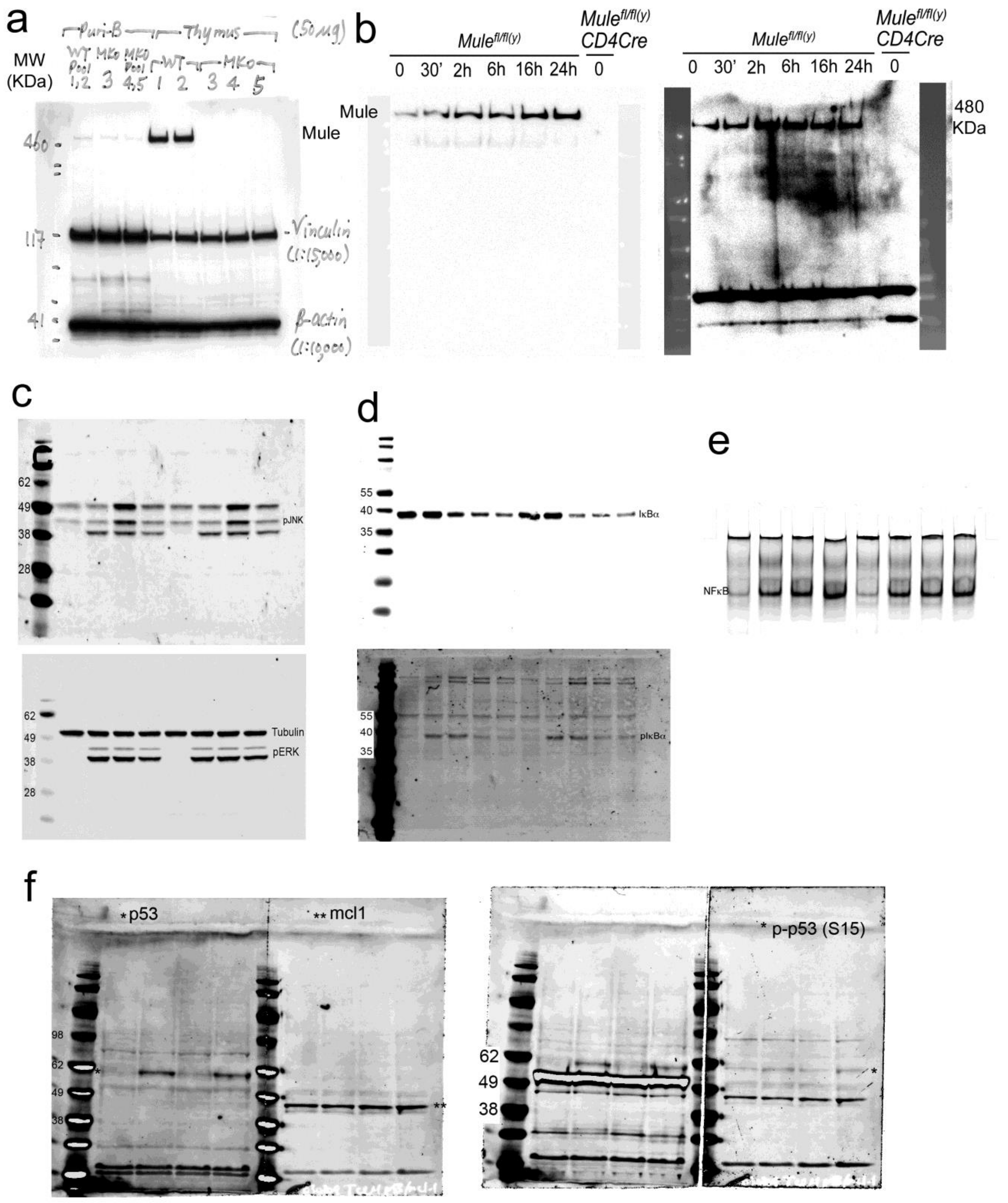

Supplementary Figure 4

Supplementary Figure 4. Whole gel images of western blots in Figures 1, 2 and 4.

(a) Figure 1b. (b) Figure 2a of CD4 cells (left) and Figure 2a of CD8 cells (right). (c) Figure 4a. (d)

Figure 4b. (e) Figure 4c. (f). Figure 4e.

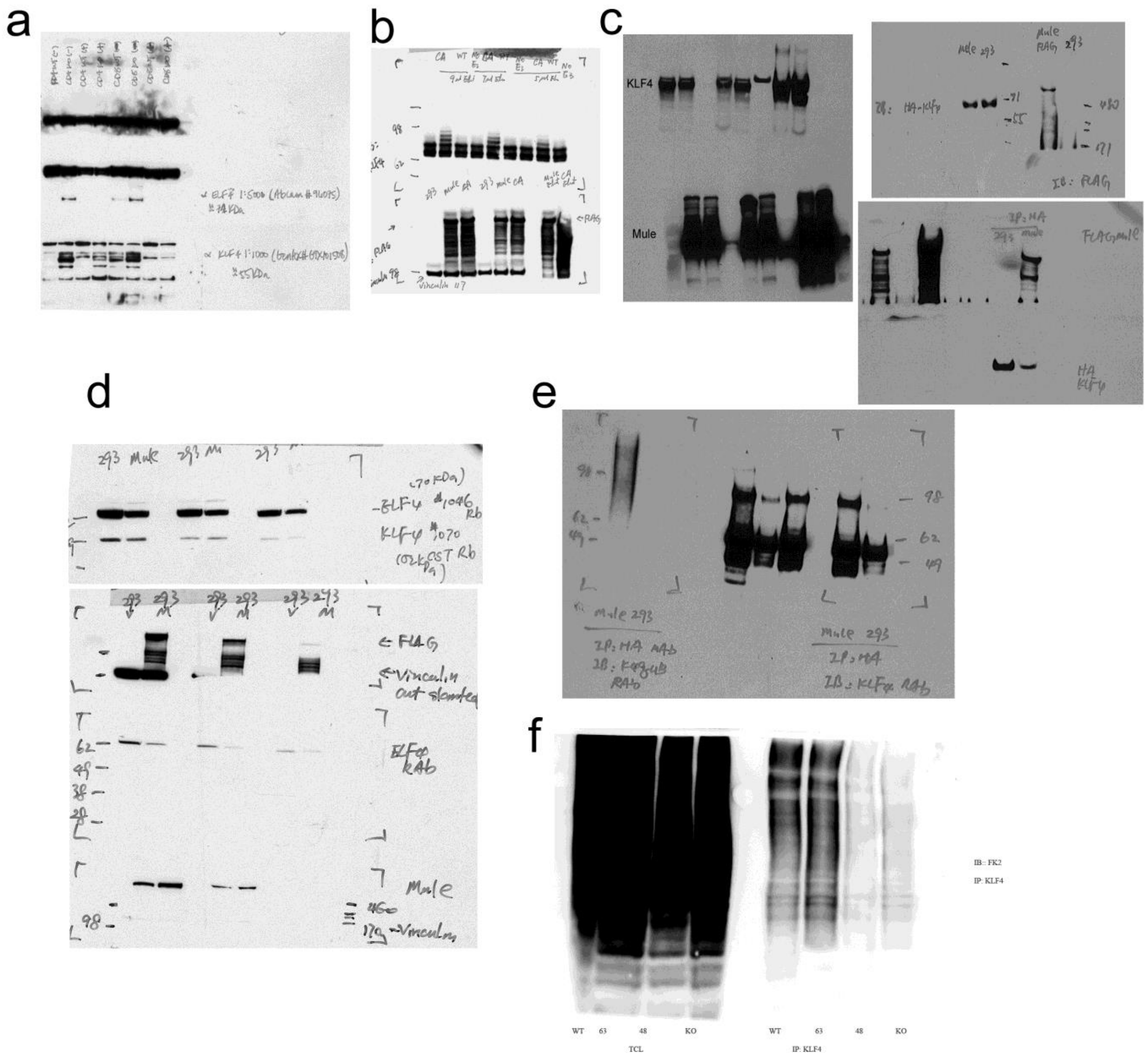

Supplementary Figure 5

Supplementary Figure 5. Whole gel images of western blots in Figures 5.

(a) Figure 5a. (b) Figure 5b. (c) Figure 5c. (d) Figure 5d. (e) Figure 5e. (f) Figure 5f.

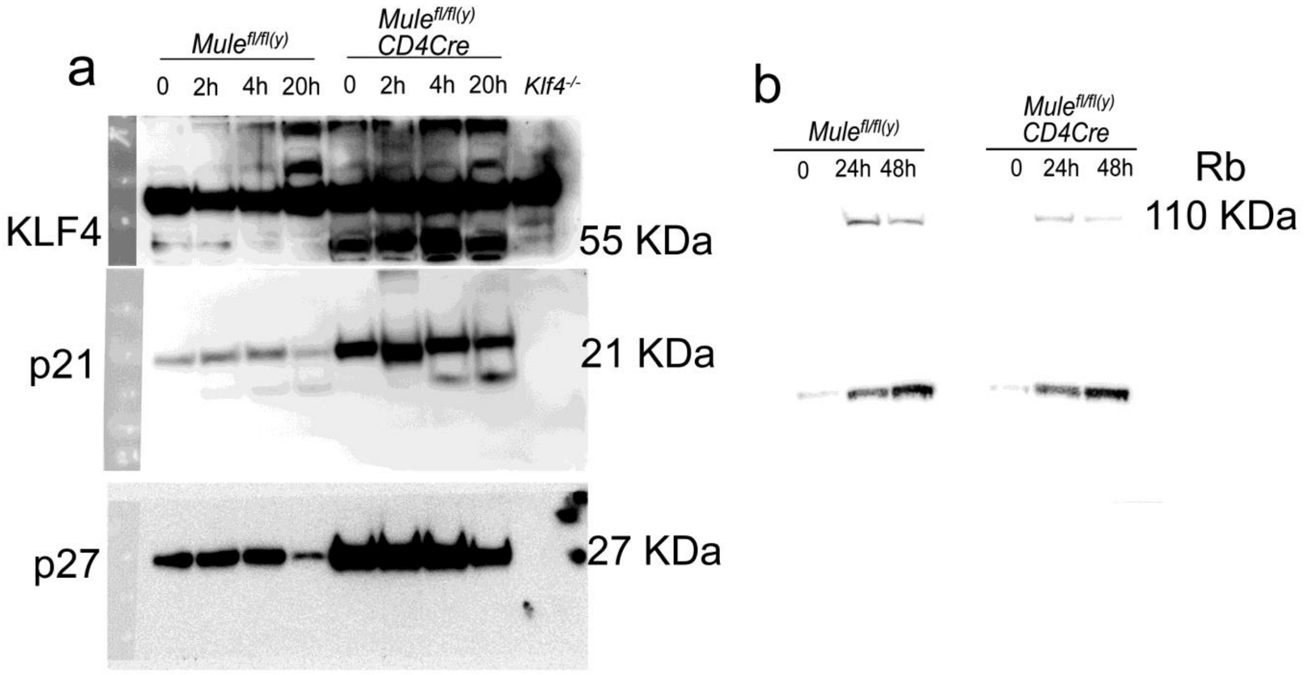

Supplementary Figure 6

Supplementary Figure 6. Whole gel images of western blots in Figures 6.

(a) Figure 6a. Lane #9 in is *Klf4* knockout T cell lysate. (b) Figure 6b.
